# A New Type of High-Sensitivity Fiber Grating Pressure Sensor

**DOI:** 10.3390/s26082490

**Published:** 2026-04-17

**Authors:** Wei-Chen Li, Wen-Fung Liu

**Affiliations:** 1 Ph.D. Program of Electrical and Communications Engineering, Feng Chia University, Taichung 40724, Taiwan; 2 Department of Electrical Engineering, Feng Chia University, Taichung 40724, Taiwan; wfliu@fcu.edu.tw

**Keywords:** fiber, fiber Bragg grating, pressure sensor

## Abstract

In this paper, we propose a high-sensitivity fiber Bragg grating (FBG) pressure sensor based on an X-shaped mechanical transducer that converts external pressure into predominantly axial strain, thereby helping to alleviate bending-dominant spectral distortion and improve measurement stability. A theoretical model is developed to describe the relationship between applied force, pressure, and grating wavelength shift. Experimental optimization was conducted by varying Ethylene Propylene Diene Monomer (EPDM) thickness, bonding materials, and contact area to achieve sensitivities of 0.291 nm/N, 0.409 nm/N, and 0.462 nm/N, respectively, within the investigated force range of 0–10 N. For measuring the under water pressure, the sensor exhibits a high sensitivity of 0.596 nm/kPa within the investigated pressure range of 0–6 kPa. The results demonstrate the nice sensing performance with high sensitivity, good linearity, and excellent repeatability. This work provides an effective approach for high-performance FBG-based pressure sensing in underwater and harsh environments.

## 1. Introduction

Pressure monitoring is essential in a wide range of engineering applications, including underwater exploration, industrial process control, structural health monitoring, and geotechnical investigation. Compared with conventional electrical sensors, optical fiber sensors offer several important advantages, such as immunity to electromagnetic interference, corrosion resistance, compact size, remote interrogation capability, and suitability for harsh environments. Among various optical sensing technologies, fiber Bragg grating (FBG) sensors have attracted extensive attention because their sensing information is wavelength-encoded, allowing stable demodulation and convenient multiplexing. However, the intrinsic pressure sensitivity of a bare fiber grating is extremely low. Xu et al. reported that the Bragg wavelength shift in an in-fiber grating under direct hydrostatic pressure was only −3.04 × 10^−3^ nm/MPa, i.e., approximately 3.04 pm/MPa [1]. This very weak pressure response makes direct pressure interrogation of a bare FBG impractical and explains why additional mechanical transduction structures are generally required. 

To enhance the pressure response of FBG sensors, one of the earliest approaches was to employ polymer-assisted packaging structures. Zhang et al. proposed a shielded polymer-coated FBG pressure sensor with a fractional wavelength sensitivity of $3.41\times 10^{-3}\;{\mathrm{MPa}}^{-1}$, which was reported to be approximately 1720 times higher than that of a bare FBG [2]. Sheng et al. later developed a lateral-pressure FBG sensor using a polymer-half-filled cylindrical structure and achieved a pressure sensitivity of $2.2\times 10^{-2}\;{\mathrm{MPa}}^{-1}$, corresponding to approximately 10,900 times the response of a bare FBG [3]. These studies clearly demonstrated that appropriate packaging can effectively convert external pressure into axial strain along the grating. Nevertheless, polymer-based structures may still suffer from viscoelasticity, long-term aging, and environmental instability, which can limit measurement repeatability and long-term reliability. 

Another widely adopted strategy is to use diaphragm-based or metallic elastic elements to amplify strain transfer. Huang et al. developed a diaphragm-type FBG pressure sensor with temperature compensation by bonding two bare FBGs directly on a circular diaphragm and achieved a sensitivity of 1.57 pm/kPa over the range of 0–1 MPa [4]. Ahmad et al. proposed a thin-metal-diaphragm FBG pressure sensor and reported a sensitivity of 0.0115 nm/psi [5]. Liang et al. further introduced a diaphragm-cantilever structure with differential wavelength demodulation and obtained a sensitivity of 339.956 pm/MPa [6]. Zhao et al. proposed a practical diaphragm-cantilever FBG pressure sensor with a sensitivity of 258.28 pm/MPa in the range of 0–2 MPa [7]. Her and Weng integrated an FBG with an epoxy diaphragm and reported pressure sensitivities of 175.5 pm/kPa, 89.5 pm/kPa, and 43.7 pm/kPa for diaphragm thicknesses of 0.5, 0.7, and 1.0 mm, respectively [8]. These studies confirm that diaphragm-type structures can substantially improve pressure sensitivity. However, many such designs still rely on bending-dominant deformation or surface strain transfer, which may introduce non-uniform strain distribution and reduce spectral stability. 

To further improve sensitivity, more advanced mechanical amplification schemes have been proposed. Schenato et al. developed a highly sensitive FBG pressure sensor based on a 3D-printed transducer and reported a pressure sensitivity of up to 240 pm/kPa, which was more than four orders of magnitude higher than that of a bare FBG [9]. Liu et al. designed a pressure sensor based on a metal diaphragm and lever structure and achieved a typical pressure sensitivity of 3.35 nm/MPa [10]. Burhanuddin et al. developed an FBG-based pore-pressure sensor utilizing a bellows system and obtained a sensitivity of 1.224 pm/kPa [11]. In another study, the same group proposed a 3-D-printed multiparameter FBG sensor for simultaneous measurement of vertical earth pressure and pore pressure, with sensitivities of 43.1550 pm/psi and 0.9841 pm/psi, respectively [12]. Liu et al. later reported a hinged-lever FBG pressure sensor with a sensitivity of 3.382 pm/kPa [13], while Hao et al. proposed a diaphragm–lever composite structure with an experimental sensitivity as high as 69.042 nm/MPa in the range of 0–0.1 MPa [14]. These studies clearly indicate that mechanical amplification is an effective route for enhancing FBG pressure sensitivity. However, lever- or diaphragm-assisted structures often require more complicated assemblies, precise mechanical alignment, or relatively large local deformation, and some designs still impose bending-dominant strain on the grating region. 

Besides FBG-based pressure sensors, interferometric and hybrid optical fiber sensors have also demonstrated remarkable pressure sensitivity. Feng et al. proposed an integrated fiber-optic sensor for simultaneous measurement of salinity, pressure, and temperature, in which the pressure channel based on Fabry–Perot interferometry exhibited a sensitivity of −9.72 nm/MPa [15]. Zhang et al. developed an optical Fabry–Perot pressure sensor for extreme deep-sea applications, achieving dual-range sensitivities of 0.125 pm/bar in the range of 0–400 bar and 0.066 pm/bar in the range of 400–1200 bar [16]. Zhao et al. proposed a high-temperature Fabry–Perot gas-pressure probe with a pressure sensitivity of −9.81 nm/MPa over 0–12 MPa and stable operation from 20 to 1100 °C [17]. In addition, Liang et al. demonstrated a Sagnac-loop twisted-pair microfiber pressure sensor encapsulated in PDMS, exhibiting an extremely high pressure sensitivity of 54.43 pm/Pa [18]. Although these interferometric or microfiber-based sensors can provide excellent pressure response, they usually require more sophisticated demodulation schemes and may be less straightforward for compact, robust, and low-cost underwater packaging than FBG-based designs.

From the above literature, it can be seen that the main challenge in FBG pressure sensing is not only to improve sensitivity, but also to maintain structural simplicity, stable strain transfer, spectral integrity, and practical applicability in harsh environments. Many existing high-sensitivity FBG pressure sensors improve performance through polymer compression, diaphragm bending, bellows deformation, or lever amplification. However, when the grating region is subjected to strong local bending or non-uniform strain distribution, the reflection spectrum may become distorted, broadened, or chirped, which can degrade measurement stability and demodulation accuracy. Therefore, a pressure transduction structure capable of converting external pressure predominantly into axial tensile strain, rather than directly imposing bending deformation on the grating region, is highly desirable. 

To provide a clearer benchmark against representative prior FBG-based pressure sensors, a comparative summary is presented in Table 1. The table includes the main structural types and key performance indicators reported in previous studies, including pressure range, sensitivity, linearity, hysteresis, and temperature compensation. This comparison helps place the proposed sensor in the context of the current state of the art and highlights the differences among polymer-packaged, diaphragm-based, bellow-based, lever-assisted, and 3D-printed transducer designs.

In this study, a novel high-sensitivity FBG pressure sensor based on an X-shaped mechanical transducer is proposed. Different from conventional bending-dominant diaphragm structures, the proposed design converts externally applied force or pressure into predominantly axial tensile strain through the rotational deformation of the X-shaped frame and the elastic compression of EPDM layers. This mechanism is expected to reduce bending-induced spectral distortion and improve strain-transfer stability. In addition, a theoretical model is established to describe the relationship between applied force, pressure, and wavelength shift, and a stepwise optimization strategy is adopted to systematically investigate the effects of EPDM thickness, bonding material, and contact area on sensor performance. The optimized structure achieved force sensitivities of 0.291 nm/N, 0.409 nm/N, and 0.462 nm/N during structural optimization, and after encapsulation for underwater operation, the pressure sensitivity reached 0.596 nm/kPa with good linearity and repeatability. These results demonstrate that the proposed X-shaped transducer provides an effective approach for realizing high-sensitivity, mechanically stable, and practically applicable FBG pressure sensing for underwater and harsh-environment applications.

## 2. Basic Principle and Sensing Head

### 2.1. Fiber Bragg Grating Sensing Principle

The operating principle of a fiber Bragg grating (FBG) is based on the Bragg condition, which relates the reflected wavelength to the effective refractive index and the grating period [19]:

(1) $$\lambda_{B}=2n_{\mathrm{eff}}\Lambda$$

where $n_{\mathrm{eff}}$ is the effective refractive index of the fiber core and Λ is the grating period. When an axial strain is applied to the FBG, both the refractive index and the grating period are altered, resulting in a shift in the Bragg wavelength.

The relative wavelength shift induced by strain can be expressed as [19]:

(2) $$\frac{\Delta \lambda_{B}}{\lambda_{B}}=\left(1-p_{e}\right)\varepsilon$$

where $p_{e}$ is the effective photoelastic coefficient and ε is the axial strain applied to the grating. Physically, $p_{e}$ represents the strain-optic response of the fiber material, that is, the extent to which the effective refractive index changes under applied mechanical strain. When axial strain is applied to the FBG, the Bragg wavelength shift arises from two simultaneous effects: the increase in grating period due to fiber elongation and the variation in effective refractive index caused by the photoelastic effect. Accordingly, $p_{e}$ quantifies the contribution of the strain-induced refractive-index change to the overall wavelength response. Therefore, the wavelength shift can be written as:

(3) $$\Delta \lambda_{B}=\lambda_{B}\left(1-p_{e}\right)\varepsilon$$

This equation indicates that the Bragg wavelength shift is directly proportional to the axial strain, which forms the fundamental sensing mechanism used in this study.

### 2.2. Pressure Sensor Fabrication

**Figure 1:**
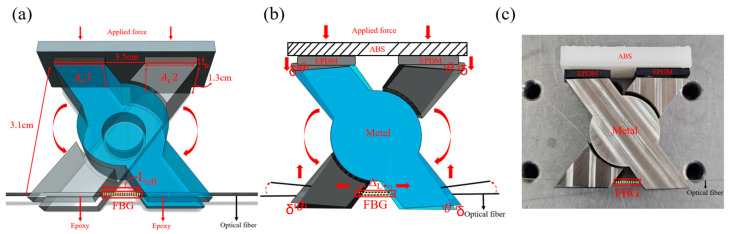
(**a**) Schematic diagram of the proposed pressure sensor. (**b**) Deformation of the X-shaped structure under applied force. (**c**) Photograph of the fabricated sensor.

The proposed sensor consists of a rigid X-shaped mechanical structure with two EPDM layers placed at the upper contact regions. Stainless steel was selected for the X-shaped transducer because of its high mechanical rigidity, structural stability, and suitability for pressure transmission. EPDM was used as an elastic buffer layer to accommodate local deformation and to provide stable force transfer to the sensing structure. The total effective contact area of the EPDM layers is defined as $A_{c}=A_{c1}+A_{c2}$, where $A_{c1}$ and $A_{c2}$ represent the effective contact areas of the two EPDM layers, respectively. The EPDM layers serve as elastic buffers to accommodate the small angular deformation (approximately 2°) of the structure under loading, thereby minimizing variation in the effective contact area and helping to ensure stable sensitivity. The fiber Bragg grating (FBG) is fixed at both ends using a two-part structural epoxy adhesive (3M™ Scotch-Weld™ DP420, 3M Company, Saint Paul, Minnesota, United States), with a bonding length of approximately 4 mm on each side. The sensor was assembled by placing the EPDM layers at the upper contact regions and fixing the FBG at both ends with the structural adhesive. This bonding configuration provides sufficient mechanical fixation while allowing the central region of the FBG to respond to the induced strain. Figure 1a shows the overall structural design of the proposed sensor, Figure 1b illustrates the deformation behavior under applied force, and Figure 1c presents a photograph of the fabricated sensor. The fabricated device is consistent with the designed X-shaped transducer configuration, confirming the practical realization of the proposed sensing head.

The detailed structural dimensions, material properties, bonding configuration, and encapsulation parameters of the proposed sensor are summarized in Table 2.

### 2.3. Mechanical Deformation and Sensitivity Model

When an external force is applied to the sensor, the load is first transmitted through the upper structure to the EPDM layers. Due to their elastic nature, the EPDM layers undergo compressive deformation, converting the applied force into vertical displacement. Assuming linear elastic behavior and uniform stress distribution, the compression displacement can be approximated according to the basic stress–strain relationship. Similar mechanically amplified modeling concepts have been reported in FBG pressure sensors with diaphragm–lever composite structures [14]:

(4) $$\delta =\frac{Ft_{p}}{E_{p}A_{c}}$$

where $t_{p}$, $E_{p}$, and $A_{c}$ represent the thickness, Young’s modulus, and effective contact area of the EPDM, respectively. Since the proposed structure contains two EPDM contact regions, the total effective contact area is defined as

(5) $$A_{c}=A_{c1}+A_{c2}$$

where $A_{c1}$ and $A_{c2}$ denote the effective contact areas of the two EPDM contact regions, respectively.

As illustrated in Figure 1b, the vertical compression of the EPDM drives the center of the X-shaped structure downward. Because of its inclined geometry, this motion is transformed into rotational movement of the arms, leading to axial elongation at both ends of the structure where the FBG is bonded. A similar displacement-transfer concept has been adopted in metal diaphragm–lever FBG pressure sensors.

This mechanism transforms vertical compression into axial elongation of the FBG. The relationship between the compression displacement and the axial displacement can be expressed as:

(6) $$\Delta L=\eta_{m}\delta$$

where $\eta_{m}$ represents the mechanical conversion factor determined by the geometry and boundary conditions of the structure. Related lever-assisted and hinged mechanical amplification concepts have also been reported in FBG pressure sensors [13].

The induced axial strain in the FBG is then given by:

(7) $$\varepsilon =\frac{\Delta L}{L_{\mathrm{eff}}}$$

Substituting the above relationships into the fundamental FBG equation, the wavelength shift can be expressed as:

(8) $$\Delta \lambda_{B}=\lambda_{B}\left(1-p_{e}\right)\frac{\eta_{m}Ft_{p}}{E_{p}A_{c}L_{\mathrm{eff}}}$$

Accordingly, the force sensitivity is given by:

(9) $$S_{F}=\lambda_{B}\left(1-p_{e}\right)\frac{\eta_{m}t_{p}}{E_{p}A_{c}L_{\mathrm{eff}}}$$

For pressure sensing, when a uniform pressure is applied over the diaphragm area A, the force can be expressed as F=PA, and the pressure sensitivity becomes:

(10) $$S_{P}=\lambda_{B}\left(1-p_{e}\right)\frac{\eta_{m}At_{p}}{E_{p}A_{c}L_{\mathrm{eff}}}$$

These results indicate that the sensitivity is governed by both the mechanical deformation process and the structural parameters, including EPDM thickness, contact area, and effective grating length.

It should be noted that the above derivation is based on a simplified first-order analytical model. The model assumes linear elastic behavior of the EPDM, approximately uniform stress distribution over the effective contact area, and a geometry-dependent mechanical conversion factor for describing the displacement transfer from vertical compression to axial elongation. Therefore, the model is mainly intended to capture the dominant sensitivity trend of the proposed structure rather than provide a rigorous full-field mechanical validation.

## 3. Experimental Set-Up and Results

### 3.1. Experimental Configuration

To evaluate the performance of the proposed sensor, experiments were conducted under both force-loading and underwater pressure conditions. In the initial stage, a push–pull force gauge (digital force gauge) was used to apply controlled external forces to the sensor for structural optimization.

**Figure 2:**
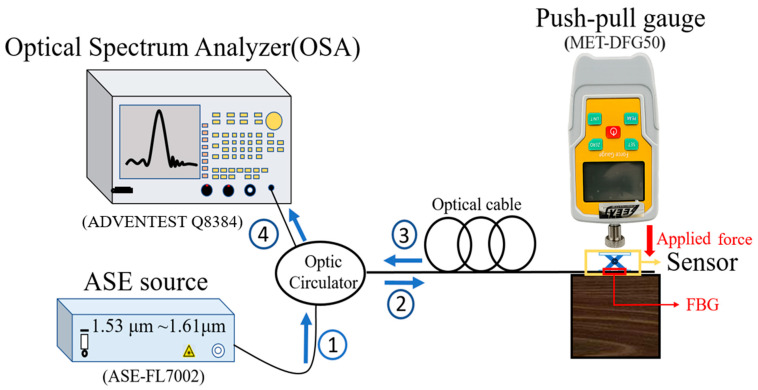
Experimental setup for force-loading calibration and sensor optimization.

As shown in Figure 2, the FBG was connected to an optical interrogation system consisting of a broadband light source (ASE-FL7002, Thorlabs, Inc., Newton, NJ, USA), an optical circulator, and an optical spectrum analyzer (Advantest Q8384, Advantest Corporation, Tokyo, Japan). The applied force was gradually increased in a stepwise manner, and the corresponding wavelength shift was recorded in real time.

**Figure 3:**
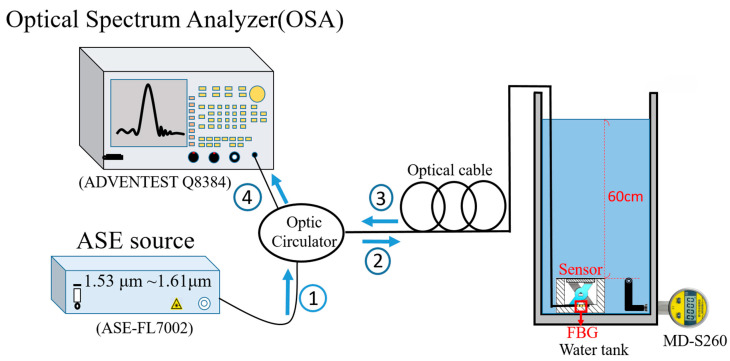
Experimental setup for underwater pressure measurement.

After completing the force-based optimization, the sensor was encapsulated and further evaluated under underwater pressure conditions. The experimental setup is illustrated in Figure 3, where the sensor was placed in a constant-temperature water tank and subjected to varying pressure levels. During the underwater test, the tank pressure was increased stepwise by controlled water injection. When the reference pressure gauge (MD-S260, accuracy: ±1%FS, Meokon Sensor Technology (Shanghai) Co., Ltd., Shanghai, China) reached each preset pressure level, the corresponding FBG wavelength was recorded using the optical spectrum analyzer. The wavelength resolution of the optical spectrum analyzer was approximately 0.01 nm. The Bragg wavelength was determined from the peak position of the reflected spectrum measured by the optical spectrum analyzer. In addition to the nominal instrument resolution, the practical wavelength consistency of the measurement system was evaluated from repeated experiments, as discussed in Section 3.7. All measurements were repeated five times under identical experimental conditions to ensure the repeatability and reliability of the experimental results. For each force or pressure level, the reported wavelength shift was taken as the mean value of five repeated measurements, and the corresponding standard deviation was used to construct the error bars shown in the calibration plots. The structural optimization experiments were conducted in a temperature-controlled environment, and the underwater pressure measurements were performed in a constant-temperature water tank. During the underwater measurements, the sensor was operated in a sealed encapsulation structure and did not directly contact the surrounding water. In addition, the water temperature was measured at the beginning and at the end of each measurement run, and no observable temperature change was recorded during the approximately 260 s test period. These observations suggest that large temperature drift during a single test was unlikely. However, continuous temperature monitoring was not performed throughout the full measurement process, and therefore the possible influence of small transient temperature fluctuations on the measured wavelength response cannot be completely excluded. The sensor was calibrated by applying known force or pressure levels and recording the corresponding Bragg wavelength shift. The sensitivities were obtained from the slope of the wavelength-shift response within the investigated operating ranges.

### 3.2. Theoretical Sensitivity Analysis

Based on the analytical model derived in Section 2.3, the theoretical sensitivity of the proposed sensor was calculated using the following parameters: Bragg wavelength $\lambda_{B}=1550\;\mathrm{nm}$, photoelastic coefficient $p_{e}=0.22$, mechanical conversion factor $\eta_{m}=1$, EPDM thickness $t_{p}=2\;\mathrm{mm}$, Young’s modulus $E_{p}=2\;\mathrm{MPa}$, effective contact area $A_{c}=2.55\;{\mathrm{cm}}^{2}$, and effective grating length $L_{\mathrm{eff}}=1\;\mathrm{cm}$. Under these conditions, the theoretical force sensitivity was calculated to be 0.474 nm/N. For pressure sensing, the effective pressure-bearing area was taken as the rectangular opening area of the upper diaphragm, which was $15\;{\mathrm{cm}}^{2}$. Based on this area, the theoretical pressure sensitivity was obtained as 0.711 nm/kPa. These theoretical values serve as first-order analytical references for comparison with the experimental results in the following sections. It should be noted that the model is based on simplified assumptions and does not include factors such as interfacial constraints, encapsulation-induced stiffness, non-uniform deformation, or possible deviations in strain transfer efficiency. Therefore, some discrepancy between the theoretical prediction and the experimental response is expected. In addition, practical fabrication deviations of the transducer may introduce additional measurement uncertainty by affecting the effective contact area, structural symmetry, and strain-transfer behavior. In the present study, repeated measurements were performed and average values were reported to reduce the influence of such practical variations.

### 3.3. Effect of EPDM Thickness

**Figure 4:**
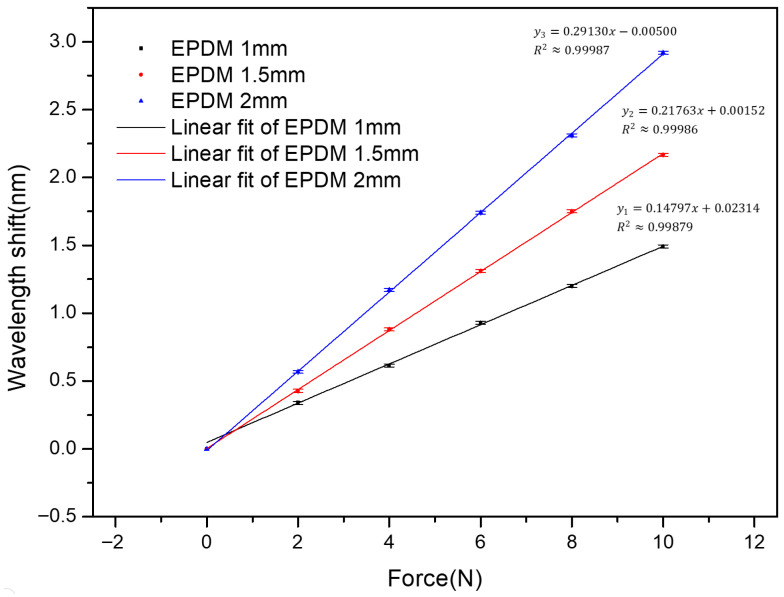
Effect of EPDM thickness on the force sensitivity of the sensor.

The effect of EPDM thickness ($t_{p}$) on sensor performance was investigated, and the results are shown in Figure 4. In this stage, only the EPDM thickness was varied, while all other parameters, including bonding condition, contact area ($A_{c}$), and structural configuration, were kept constant. According to the sensitivity expression given in Equation (9), the force sensitivity is proportional to the EPDM thickness $t_{p}$, indicating that $t_{p}$ plays a key role in governing the mechanical deformation and subsequent strain transfer to the FBG. Therefore, for EPDM thicknesses of 1 mm, 1.5 mm, and 2 mm, the theoretical sensitivity ratio is expected to follow a proportional relationship of 1:1.5:2. The experimental results show that the sensitivity increases with increasing EPDM thickness, reaching 0.291 nm/N under the tested conditions. The measured sensitivity ratios generally follow the proportional trend predicted by Equation (9), confirming that the EPDM layer plays a dominant role in governing the mechanical deformation and strain transfer behavior of the sensor. However, the experimental results do not exhibit perfectly linear scaling. This deviation can be attributed to non-uniform stress distribution, structural constraints, and partial loss of strain transfer efficiency within the EPDM layer. In this study, a stepwise optimization strategy was adopted, in which the EPDM thickness was first optimized and the corresponding optimal value was used as the baseline for the subsequent investigations. The same procedure was applied to the following experiments on bonding material and contact area, where only one parameter was varied at a time while all others were kept unchanged, thereby ensuring a consistent and systematic evaluation of sensor performance.

### 3.4. Effect of Bonding Material

**Figure 5:**
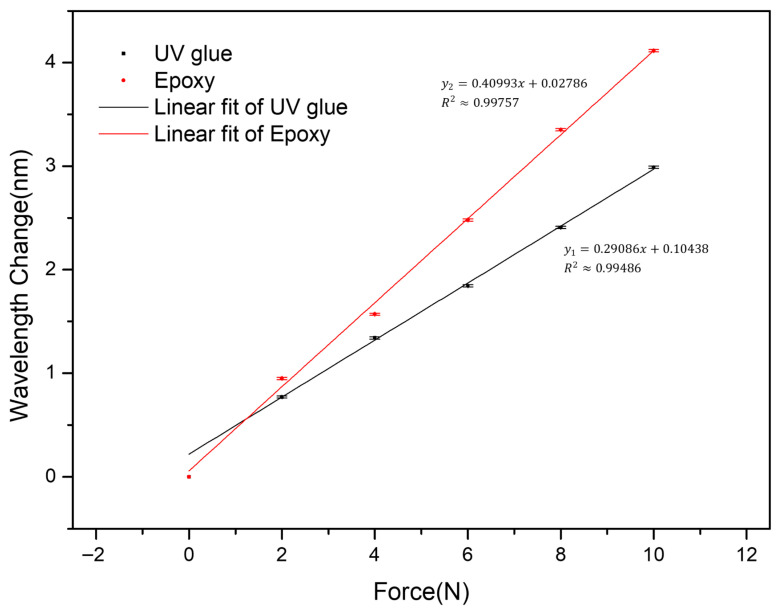
Effect of bonding material on the force sensitivity of the sensor.

Following the stepwise optimization approach described in Section 3.3, this subsection further investigates the effect of the bonding material on the sensor performance, as shown in Figure 5. The comparison in this stage was conducted using the optimal EPDM thickness obtained in Section 3.3, while all other parameters were kept unchanged. Under this condition, the UV adhesive serves as the baseline bonding configuration used in the previous stage, and its corresponding sensitivity remains consistent with the results obtained in Section 3.3. By contrast, replacing the UV adhesive with Epoxy leads to increased sensitivity, reaching 0.409 nm/N under the optimized bonding condition. This indicates that the improvement observed in this stage is mainly attributed to the change in bonding material rather than the EPDM thickness. It should be noted that the bonding material is not explicitly included in Equation (9). Nevertheless, it influences the strain transfer condition between the mechanical structure and the FBG. Different adhesives result in variations in interfacial stiffness and bonding effectiveness, which in turn affect the effective axial strain applied to the grating. Therefore, although the bonding effect is not directly reflected in the simplified analytical model, the experimental results demonstrate that it is a critical factor in practical sensitivity optimization. In the present design, the Epoxy provides better mechanical coupling than the UV adhesive, leading to an enhanced sensor response.

### 3.5. Effect of Contact Area

**Figure 6:**
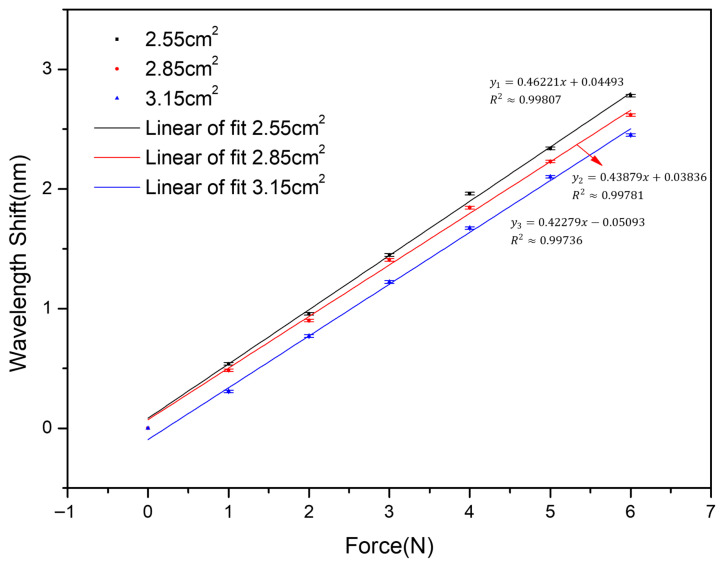
Effect of contact area on the force sensitivity of the sensor.

The effect of effective contact area on the sensor performance was further investigated, and the results are shown in Figure 6. Following the stepwise optimization approach described in the previous sections, this stage was conducted using the optimal EPDM thickness and bonding condition, while only the contact area was varied. In this study, the effective contact area $A_{c}$ was adjusted by modifying the top contact geometry, with values of 3.15 cm^2^, 2.85 cm^2^, and 2.55 cm^2^, respectively. According to the sensitivity expression given in Equation (9), the force sensitivity is inversely proportional to the effective contact area. Therefore, the sensitivity ratio is expected to follow the inverse relationship of the contact area. The experimental results show that the sensitivity increases as the contact area decreases, reaching 0.462 nm/N at $A_{c}=2.55\;{\mathrm{cm}}^{2}$. As the contact area decreases from 3.15 cm^2^ to 2.55 cm^2^, the corresponding sensitivity exhibits a clear increasing trend. The relative variation in sensitivity generally follows the inverse proportional relationship predicted by Equation (9), although slight deviations are observed due to practical factors. These deviations can be attributed to non-uniform stress distribution and structural constraints in the practical configuration. Nevertheless, the overall agreement between the experimental results and the analytical prediction supports the inverse relationship between sensitivity and contact area. The maximum sensitivity reaches 0.462 nm/N, which is in reasonable agreement with the simplified theoretical prediction of 0.474 nm/N. This result suggests that the proposed stepwise optimization approach effectively improves the sensor performance and that the experimental response is generally consistent with the expected trend of the analytical model. 

**Figure 7:**
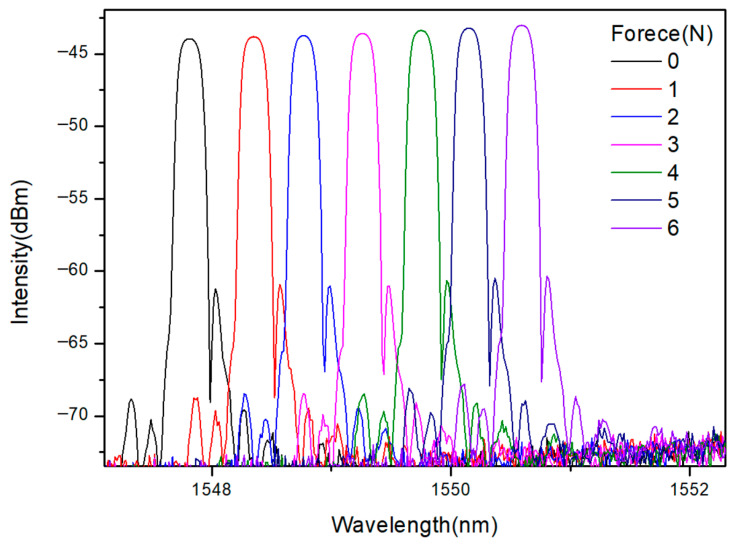
Representative reflection spectra of the optimized FBG pressure sensor with an effective contact area of 2.55 cm^2^ under different applied force levels.

To further examine the spectral response of the optimized sensor, representative reflection spectra of the FBG under different applied force levels were recorded for the configuration with an effective contact area of 2.55 cm^2^, as shown in Figure 7.

The results show that the reflection spectrum mainly undergoes a progressive wavelength shift with increasing applied force, while the overall spectral profile remains generally stable within the investigated range. No obvious peak splitting or severe spectral distortion was observed. These results provide qualitative spectral evidence that the proposed X-shaped transducer mainly converts the applied load into axial tensile strain on the FBG and helps reduce bending-dominant spectral distortion under the present experimental conditions. Based on this optimized configuration, the sensor is subsequently encapsulated and evaluated under underwater pressure conditions. 

### 3.6. Underwater Pressure Measurement

**Figure 8:**
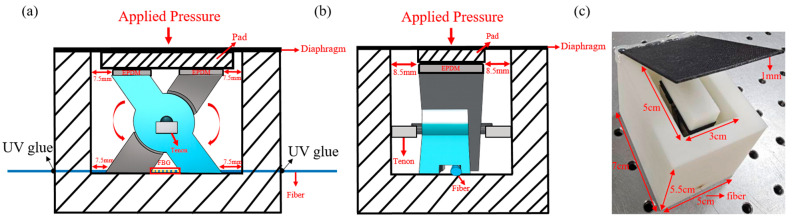
(**a**) Front view of the encapsulated underwater pressure sensor. (**b**) Side view of the encapsulated underwater pressure sensor. (**c**) Photograph of the fabricated sensor.

**Figure 9:**
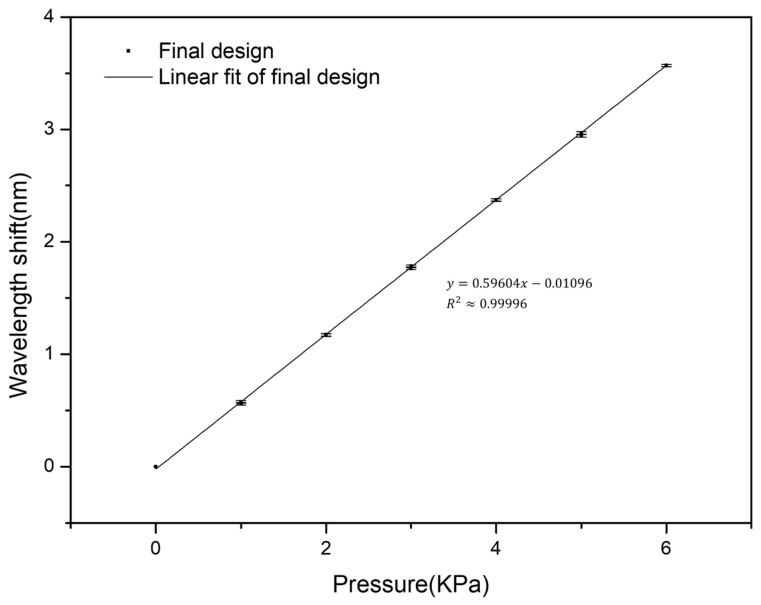
Pressure response of the sensor under underwater conditions.

Based on the optimized configuration obtained through the stepwise optimization process, the sensor was encapsulated and evaluated under underwater pressure conditions. In the encapsulated design, the external water pressure was applied to the sensing structure through the rectangular opening area of the upper diaphragm, which measured 5 × 3 cm, corresponding to an effective pressure-bearing area of 15 cm^2^. The upper diaphragm of the underwater packaging structure was formed by covering the 15 cm^2^ pressure-receiving opening with a 1 mm thick ABS plate. This diaphragm was mainly used to isolate the sensing structure from direct contact with water while maintaining a defined pressure-receiving area. Waterproof adhesive and hot-melt adhesive were applied to seal the joints of the encapsulation structure. Although no obvious water ingress was observed during the present underwater experiments, the influence of the diaphragm layer on pressure-transfer efficiency and measurement accuracy was not separately quantified in this study and remains for future investigation. The applied water pressure was transmitted through the diaphragm to the sensing structure, and the entire underwater packaging structure was fully sealed during the measurement. The experimental setup is shown in Figure 8, where Figure 8a illustrates the front view of the encapsulated sensor, showing the overall structural layout, Figure 8b presents the side view highlighting the layered configuration and thickness of the sensing structure, and Figure 8c shows a photograph of the fabricated and encapsulated sensor, confirming the practical implementation of the proposed design. The measured pressure response is presented in Figure 9. The experimental results show that the sensor exhibits a linear response to the applied pressure, with a measured pressure sensitivity of 0.596 nm/kPa. This value is in reasonable agreement with the theoretical prediction of 0.711 nm/kPa derived from Equation (10), although some deviation is observed in practice. The slight deviation between the experimental and theoretical results can be attributed to practical factors such as encapsulation effects, structural constraints, and non-uniform pressure distribution. In addition, since FBG sensors are intrinsically sensitive to both strain and temperature, the present underwater measurements were performed in a constant-temperature water tank without additional temperature compensation. Considering that the sensor was enclosed in a sealed structure, that no observable water-temperature change was recorded between the beginning and the end of each approximately 260 s measurement run, and that the sensing element did not directly contact the surrounding water, significant temperature drift during a single test was considered unlikely. However, because continuous temperature monitoring was not performed, small transient temperature fluctuations cannot be completely excluded. Nevertheless, the overall agreement shows general consistency with the simplified analytical prediction and demonstrates that the optimized sensor structure can effectively convert pressure into measurable optical signals. These results indicate that the proposed sensor, combined with the stepwise optimization approach, achieves high sensitivity and reliable performance, making it suitable for underwater pressure sensing applications. 

### 3.7. Underwater Pressure Sensor Repeatability

**Figure 10:**
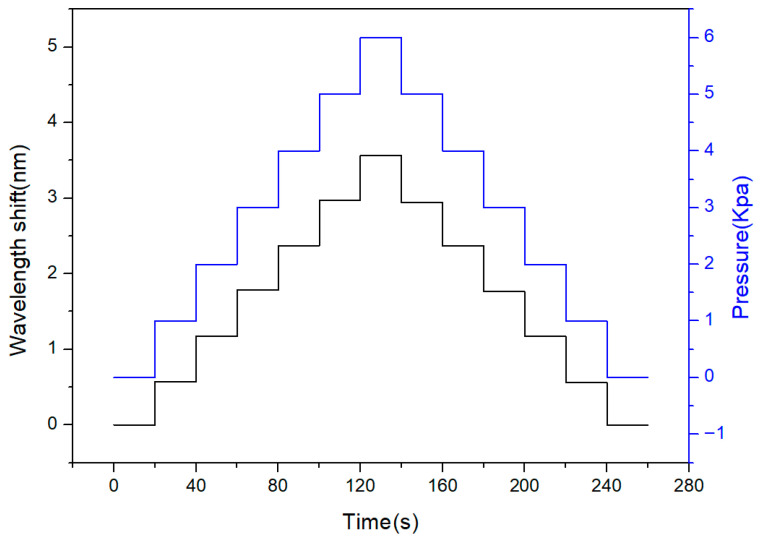
Cyclic pressure response of the sensor under underwater conditions. The black line represents the measured wavelength shift, while the blue line represents the applied pressure profile during the loading and unloading processes.

The short-term cyclic repeatability of the encapsulated underwater pressure sensor was evaluated through cyclic pressure loading tests, and the results are shown in Figure 10. In this experiment, the sensor was subjected to stepwise pressure variations under underwater conditions. The applied pressure was increased and then decreased in a cyclic manner, with each pressure level maintained for approximately 20 s to ensure stable measurement. The corresponding wavelength shift was recorded simultaneously. In Figure 10, the black curve represents the measured wavelength shift, whereas the blue curve represents the applied pressure profile during the loading–unloading cycle. The pressure and wavelength responses exhibit a consistent stepwise trend, indicating good short-term repeatability under cyclic loading. The wavelength variation closely follows the applied pressure profile during both loading and unloading processes, demonstrating good linearity. The hysteresis error, calculated from the maximum difference between the loading and unloading responses at the same pressure level and normalized to the full-scale output, was approximately 0.76%FS. All experimental data presented in this study, including the calibration results shown in Figure 4, Figure 5, Figure 6 and Figure 9, represent the mean values obtained from five repeated measurements under identical conditions. The corresponding error bars in these calibration figures denote ±1 standard deviation. The standard deviation of the wavelength variation was approximately 0.011 nm, indicating good measurement consistency during the present cyclic test. Based on the measured pressure sensitivity of 0.596 nm/kPa, this corresponds to an estimated pressure resolution of approximately 0.018 kPa under the present experimental conditions. These results confirm that the proposed sensor exhibits good short-term repeatable performance under cyclic underwater pressure conditions. However, a more comprehensive evaluation of long-term stability, including drift, creep, and multi-cycle durability, remains for future work.

## 4. Conclusions

In this study, a novel fiber Bragg grating (FBG)-based pressure sensor was proposed and experimentally demonstrated. The sensor employs a mechanical structure combined with EPDM and an optimized bonding configuration to effectively convert external pressure into axial strain, enabling high-sensitivity optical sensing. A stepwise optimization approach was adopted to systematically investigate the effects of EPDM thickness, bonding material, and contact area on sensor performance. The results show that the sensitivity increases with EPDM thickness, is significantly influenced by bonding conditions, and follows an inverse relationship with the effective contact area, consistent with the analytical model. Under the optimized configuration, the sensor achieves a force sensitivity of 0.462 nm/N, which is in reasonable agreement with the simplified analytical prediction.

In addition, representative reflection spectra under different applied force levels showed that the sensor response was mainly characterized by wavelength shift while maintaining a generally stable spectral profile within the investigated range. When applied to underwater pressure sensing, the measured pressure sensitivity reaches 0.596 nm/kPa, and the experimental response is generally consistent with the trend predicted by the analytical model. The analytical model therefore provides useful first-order guidance for structural design and parameter optimization, although it does not represent a rigorous full mechanical validation. In addition, cyclic pressure tests demonstrate good repeatability, short-term cyclic performance, and minimal hysteresis. These results indicate that the proposed sensor design, together with the stepwise optimization strategy, enables high sensitivity and reliable performance. It should also be noted that the present work represents a preliminary experimental demonstration of the proposed sensor architecture. Although the optimization experiments were carried out in a temperature-controlled environment and the underwater measurements were conducted in a constant-temperature water tank, no observable change in water temperature was recorded between the beginning and the end of each underwater measurement run. However, because continuous temperature monitoring was not performed throughout the full measurement process, the possible influence of small transient temperature fluctuations on the measured response cannot be completely excluded. Therefore, a systematic investigation of temperature cross-sensitivity and long-term thermal stability remains for future study. The developed sensor shows strong potential for underwater pressure monitoring and related sensing applications.

## Figures and Tables

**Table 1:** Comparison of representative prior FBG-based pressure sensors and the proposed sensor.

Ref.	Structure/Type	Pressure Range	Sensitivity	Linearity	Hysteresis	Temperature Compensation
[2]	Shielded polymer-coated FBG	0–0.44 MPa	5.28 pm/kPa	99.92%	NR	No
[3]	Polymer-half-filled metal cylinder	0–0.2 MPa	33.876 pm/kPa	99.909%	NR	No
[4]	Diaphragm-type	0–1 MPa	1.57 pm/kPa	99.996%	NR	Yes
[6]	Diaphragm-cantilever	0–10 MPa	0.339 pm/kPa	99.997%	NR	Yes
[7]	Diaphragm-cantilever	0–2 MPa	0.258 pm/kPa	99.9%	NR	Yes
[8]	Epoxy diaphragm	0.5–3 kPa	175.5 pm/kPa	99.99%	NR	No
[9]	3D-printed transducer	0–115 kPa	240 pm/kPa	NR	NR	Yes
[10]	Diaphragm–lever	0–0.5 MPa	3.35 pm/kPa	99.84%	NR	Yes
[11]	Bellow-based	0–60 kPa	1.224 pm/kPa	99.41%	NR	Yes
[13]	Hinged-lever	0–1 MPa	3.382 pm/kPa	99.99%	NR	Yes
[14]	Diaphragm–lever composite	0–0.1 MPa	69.042 pm/kPa	99.9%	0.623%	Yes
[15]	Hybrid optical sensor	0–0.5 MPa	−9.72 pm/kPa	99.95%	NR	Yes
This work	X-shaped transducer	0–6 kPa	596 pm/kPa	99.996%	0.76%FS	No

**Table 2:** Summary of the structural, material, bonding, and encapsulation parameters of the proposed sensor.

Parameter	Value
X-shaped transducer material	Stainless steel
Fabrication route	Stainless-steel casting process
Overall dimensions of the transducer	3.5 × 3.1 × 1.3 cm
EPDM thickness	2 mm
EPDM dimensions	$A_{c1}$: 1 × 1.25 cm; $A_{c2}$: 1 × 1.3 cm
EPDM Young’s modulus	2 MPa
Fiber type	Single-mode fiber
FBG length	1 cm
Coating condition	Coating removed in the FBG sensing region
Adhesive type	3M™ Scotch-Weld™ DP420 structural epoxy adhesive
Bonding length on each side	4 mm
Overall dimensions after encapsulation	7 × 5.5 × 5 cm
Upper pressure-receiving opening dimensions	5 × 3 cm
Effective pressure-bearing area	15 cm^2^

## Data Availability

The original contributions presented in this study are included in the article. Further inquiries can be directed to the corresponding author.
